# Injured Self: Autobiographical Memory, Self-Concept, and Mental Health Risk in Breast Cancer Survivors

**DOI:** 10.3389/fpsyg.2020.607514

**Published:** 2020-11-04

**Authors:** Valeria Sebri, Stefano Triberti, Gabriella Pravettoni

**Affiliations:** ^1^Department of Oncology and Hemato-Oncology, University of Milan, Milan, Italy; ^2^Applied Research Division for Cognitive and Psychological Science, IEO European Institute of Oncology IRCCS, Milan, Italy

**Keywords:** breast cancer, self, self memory system, autobiographical memory, injured self

## Introduction

Chronic diseases do not only affect the body (Trusson et al., 2016). Research shows that when one is delivered a diagnosis to manage over the course of future life, their self-concept is altered in two main ways. First, one's own body is felt as the source of danger, fear, and “betrayal”: interoceptive sensations and pain once ignored or easily dismissed suddenly become salient and disruptive, because they could be related to threats to one's own life and safety (Harris et al., 2017). For example, women who have fought breast cancer could find themselves performing “checking behaviors” (i.e., touching sensitive areas of their own bodies looking for nodules or anomalies) compulsively over a typical day (McGinty et al., 2016).

Second, one's own identity should be reframed as that of a patient, with notable consequences on everyday life activities, self-management and lifestyle (McGannon et al., 2016; Sebri et al., 2019). For example, one should rethink everyday activities and expectations for the future, taking health management into account. Breast cancer survivors deal with the implications of their changed self-identity (e.g., the perception of being healthy women, empowered survivors, or women at risk) (Gibson et al., 2014, 2015). The illness experience may disrupt people's lives, for example in terms of relationships, priorities, sense of time, and values (Marzorati et al., 2017). Moreover, treatments (e.g., chemotherapy, hormonotherapy, radiation therapy, surgery) lead to notable physical outcomes such as breast removal, skin discoloration, hair loss, and sexual dysfunctions and to distortions in body image as a consequence (Triberti et al., 2019b). Such outcomes lead to modification of both self-narrative and bodily self-representations (Serletti et al., 2011; Yang et al., 2017). This way, one's identity as a woman is affected by storylines articulated around the relationship between self-representations and autobiographical memories as a patient and/or a survivor (Conway and Pleydell-Pearce, 2000; McGannon and Spence, 2010, 2012; Nieto et al., 2019).

This closely-knit and reciprocal interconnection between the Self and autobiographical memories is conceptualized as the Self-Memory System (SMS) (Conway, 2005), a process with long-lasting effects on individuals' attitude and behaviors (Giffard et al., 2013; Morel et al., 2015). The Self modulates the access to long-term knowledge (Conway, 2005) influencing mood states and meaning making of life events (Henry et al., 2010; Vos, 2015; Franco et al., 2017). For this reason, the flexibility of SMS is essential to reach individual goals of emotion regulation in an appropriate and calibrated manner thanks to the healthy association between self-representations and autobiographical memory (especially the self-defining ones) (Josephson et al., 1996). Indeed, Self-defining memories are a specific type of autobiographical memory that is characterized by vividness, affective intensity, linkage to similar memories, high level of rehearsal, and connection to concern or unresolved conflict (Singer and Moffitt, 1991–1992; Singer and Salovey, 1993). The inability to experience a correspondence between self-representations, autobiographical memories and real-life experiences leads to the destruction of any delineated sense of Self (Conway et al., 2004).

## The Self-Memory System in Breast Cancer Patients

The experience of breast cancer has important consequences on mental health, many of them mediated by the alterations within the SMS. Literature shows high levels of anxiety, depression, and distress until some years after acute treatments (Woertman and Van den Brink, 2012) as well as possible general symptoms of psychopathology (Burgess et al., 2005; Gandubert et al., 2009). Up to one third of breast cancer patients may suffer from psychological adjustment due to surgery and adverse effects of adjuvant cancer treatments (Fafouti et al., 2010). For example, Nieto et al. (2019) underline that intrusive memories of cancer experiences and avoidance related to such events are associated with autobiographical memory issues, that is a feature of depressive thinking. This suggests that the experience of disease and treatments may give rise to psychopathological outcomes such as depression, anxiety, intense feeling of worry, and stress linked to emotional turmoil (Hamer et al., 2009; Lucchiari et al., 2016) associated with illness self-schemas. These illness-related events affect memory because illness-relevant information becomes more salient and accessible than others (Clemmey and Nicassio, 1997), for example greater identification as a cancer survivor as well as recent cancer treatment contribute to predict maladaptive autobiographical thinking processes (Sansom-Daly et al., 2018). Moreover, in chronic illness, the self-schemas constantly influence the organization of symptoms perception and behaviors by affecting the general patient's psychological well-being. On the contrary, illness self-schemas in acute illness (e.g., upset stomach or headache) are activated temporarily by the occurrence of time-limited conditions (Clemmey and Nicassio, 1997).

We argue that breast cancer survivors have to deal with a renovated overall Self with the integration of self-representations based on the autobiographical memory of the oncological experience. This added self-representation, an “Injured Self,” involves all breast cancer patients and their long-term Self that is interconnected with the episodic memory system (Conway, 2005). In a breast cancer experience especially, individuals who identify themselves with illness-schemas behave in a manner consistent with this self-definition (Clemmey and Nicassio, 1997). As a consequence, women after breast cancer have to recognize and manage a new self-representation that affects their attitudes and behaviors by specific emotions and with possible psychopathological outcomes. Specifically, according to Giffard et al. (2013), even experience-specific self-representations are associated with the “working self” or the ongoing expression of one's own identity in actions and behavior. The working self and working memory are interdependent with one another, and the former is strictly related to values, aims, beliefs, and desires as well (Giffard et al., 2013). In this sense, the Injured self could be “added” to that self-representation that actively influences everyday life decisions and activity, in an event that should be considered critical in memory formation (Zacks et al., 2001). For example, a patient may abandon an activity she likes just because she considers herself weak and ill. However, an Injured Self could lead to self-discrepancy phenomena. The self-discrepancy theory by Higgins (1987) suggests that there may be discrepancy among multiple self-representations. The higher the discrepancy is, the higher mood diseases and psychopathological outcomes may emerge. This theory is supported by large evidence in breast cancer patients and survivors; breast cancer treatments can increase the discrepancy between how one actually is and how one would like to be and appear, as it emerged from a research where breast cancer survivors were invited to create digital avatars to represent themselves (Triberti et al., 2019a).

Breast cancer patients and survivors need to integrate various self-schemas into a coherent one. For this aim, the episodic memory system provides input to the working self and includes some knowledge in autobiographical memory influenced by goal-relevance (Conway, 2005). This way, the working self is particularly important to create appropriate images of the Self following the self-coherence request (Markus and Nurius, 1986). During memory construction, the working self is indeed the moderator between the demands of memory (that corresponds to reality and actual experiences) and coherence (memory that should be consistent with one's current self-images, beliefs, and aims) (Conway et al., 2004).

## The Main Consequences of the Injured Self

We maintain that six areas relevant to health and mental health management are affected by an Injured Self representation (see Table 1):

- *adherence to treatments*: the current literature evidences the relevance of patients' active role in their own care and treatment (Kondylakis et al., 2017). Patient adherence to prescribed intervention can be influenced by the type of physical intervention proposed (Condorelli and Vaz-Luis, 2018) and by individual characteristics (Castellano-Tejedor et al., 2015). For example, depressed patients lack engagement in health behaviors (Kaplan et al., 2010). Similarly, a self-representation rich in negative mood states as the Injured Self in oncological patients may lead people to not accept this new role removing the idea of being patients and consequently to adhere to treatments in a passive and lacking manner, which would be evident when they struggle or refrain from actively taking decisions;
- *view on the future*: patients with positive future representations of themselves are more able to adopt adaptive coping styles useful to face the disease (Zhang et al., 2010). Yet, breast cancer patients may develop fear to think and dream about the future, being focused on their self-representation as patients only (Koch-Gallenkamp et al., 2016; Sansom-Daly et al., 2018). This way, structured goal setting is seriously limited in patients because of an overall perception of hopelessness grounded in the self-representation;
- *daily lifestyle and relationships*: daily life requests changes during oncological experience in terms of routine, relationships, and the lack of independence (Jacobs et al., 2018). As consequence, breast cancer patients and survivors might remain trapped in the idea of being patients who are not self-sufficient; on the contrary, they could want to demonstrate their willingness to be healthy, stressing this aspect every day;
- *emotion regulation*: mood disorders (e.g., anxiety, depression, and distress) are connected with impairments in autobiographical memories and self-representations of breast cancer patients and survivors (Giffard et al., 2013). Being unaware of one's own inner sensations and feeling decreases the ability to attenuate emotional arousal leading to disrupted self-regulation emotions (Herwig et al., 2010);
- *coherence and flexibility of the overall Self* : breakdown in life continuity affects self-identity making the cancer event an important part of self-representations (Cheung and Delfabbro, 2016). Identifying as a cancer patient or survivor can have positive effects on both mental and physical well-being whether integrated in a total and coherent idea of the Self.

**Table 1:** Injured Self's features and their consequences for mental health and health management.

	**The Injured Self's features**	**Consequences**	**Therapeutic aims**
Adherence	See themselves only as patients without autonomy	- Rejection of oncological treatments; - Accept treatments passively	Positive adherence to treatments as personal choice
Future	Perception of hopelessness and absence of coping resources	- Lack of dreams and future projects	Start to think to the future in terms of new possibilities and challenges
Relations	Fear of not being always self-sufficient	- Stress the desire to show our healthy physical and psychological status in front of the others	Knowing one's own limits; being autonomous while recognizing to need care at the same time
Emotions	Anxiety, depression, and distress	- Poor Quality of Life; - Difficulties in emotion regulation	Balance among positive and negative emotions by introspection with awareness for inner feelings
Self	Experience of self-fragmentation	- Difficulties in the integration of the Injured Self into the overall Self	Connect and integrate this new self-representation; stress its positive outcomes

To this end, literature reports both individual and group psychological interventions to assess and face psychological well-being in breast cancer patients and survivors. Nowadays, many psychological interventions are based on the cognitive-behavioral approach with added physical exercises (Fong et al., 2012; Benton et al., 2014) and arts/dancing therapies (Björneklett et al., 2013). We argue to propose structured psychological intervention able to face with the interconnection of autobiographical memories and self-representation over breast cancer experience specifically. Furthermore, studies suggest that the various vulnerabilities of a breast cancer survivor should be followed up by mental health professionals to recognize and timely treat any mental disease that may appear (Fafouti et al., 2010).

## Conclusions

The experience of chronic illness may affect one's own self-representation deeply. Not only should self-representations suddenly include features such as being a patient, weak and frail, and in constant need of support, but also the Self-Memory System is threatened by signals coming from interoceptive and exteroceptive perception of the body (e.g., internal feelings or seeing oneself in the mirror). Furthermore, prospective thinking and goal setting, abilities necessary for imagining and pursuing one's own personal future, are severely altered. Breast cancer is a disease and illness where such process is particularly evident, since patients are affected by “injuries” to the self even after they received successful treatment. Indeed, treatments themselves may bring about notable consequences for quality of life and self-perception. The present study aimed at describing how an “Injured Self” develops in breast cancer patients as illness representation and its consequences on perceptions, emotions, and adherence to treatments. According to literature, several daily difficulties could be associated with the Injured Self in terms of psychopathological outcomes and experience of self-fragmentation with negative influences on behaviors. Psychological interventions should aim to reintegrate self-coherence working also on the Injured Self in reference to time of appearance, one's individual changes, and the overall and subjective interpretation of the events. For example, literature reports on the efficacy of life review therapy in cancer patients, based on supporting the patients to retrieve positive memories, re-evaluate life events, and re-construct patients' life narrative (Kleijn et al., 2018). Future research should still explore what are the other characteristics associated with chronic illness-related alterations to the self as well as the appropriate psychological treatment. It is also possible that such concept could be adapted to other chronic pathological conditions, focusing on the specificities of any disease in terms of experience, salient memory, and continual construction of one's own self-representation.

## Author Contributions

VS conceived the ideas presented in the article and wrote the first draft. ST contributed with discussion on the ideas presented and edited the article. GP contributed with important intellectual contents and supervised the whole process. All authors contributed to the article and approved the submitted version.

## Conflict of Interest

The authors declare that the research was conducted in the absence of any commercial or financial relationships that could be construed as a potential conflict of interest.
